# Evidence for the different physiological significance of the 6- and 2-minute walk tests in multiple sclerosis

**DOI:** 10.1186/1471-2377-12-6

**Published:** 2012-03-01

**Authors:** Robert W Motl, Yoojin Suh, Swathi Balantrapu, Brian M Sandroff, Jacob J Sosnoff, John Pula, Myla D Goldman, Bo Fernhall

**Affiliations:** 1 Department of Kinesiology and Community Health, University of Illinois at Urbana Champaign, 233 Freer Hall, Urbana, IL 61801, USA; 2 School of Medicine, University of Illinois at Peoria, Peoria, IL 61603, USA; 3 Department of Neurology, University of Virginia, Charlottesville, VA 22908, USA; 4 College of Applied Health Sciences, University of Illinois at Chicago, Chicago, IL 60612, USA

**Keywords:** Ambulation, Energy expenditure, Multiple sclerosis, Oxygen consumption, Walking

## Abstract

**Background:**

Researchers have recently advocated for the 2-minute walk (2MW) as an alternative for the 6-minute walk (6MW) to assess long distance ambulation in persons with multiple sclerosis (MS). This recommendation has not been based on physiological considerations such as the rate of oxygen consumption (V·O_2_) over the 6MW range.

**Objective:**

This study examined the pattern of change in V·O_2 _over the range of the 6MW in a large sample of persons with MS who varied as a function of disability status.

**Method:**

Ninety-five persons with clinically-definite MS underwent a neurological examination for generating an Expanded Disability Status Scale (EDSS) score, and then completion of the 6MW protocol while wearing a portable metabolic unit and an accelerometer.

**Results:**

There was a time main effect on V·O_2 _during the 6MW (*p *= .0001) such that V·O_2 _increased significantly every 30 seconds over the first 3 minutes of the 6MW, and then remained stable over the second 3 minutes of the 6MW. This occurred despite no change in cadence across the 6MW (*p *= .84).

**Conclusions:**

The pattern of change in V·O_2 _indicates that there are different metabolic systems providing energy for ambulation during the 6MW in MS subjects and steady state aerobic metabolism is reached during the last 3 minutes of the 6MW. By extension, the first 3 minutes would represent a test of mixed aerobic and anaerobic work, whereas the second 3 minutes would represent a test of aerobic work during walking.

## Background

Walking performance has been identified as a key outcome variable in clinical trials and practice involving persons with multiple sclerosis (MS) [1]. This is based on its association with disease progression [2] and value as an important personal function [3] with profound implications for independence, quality of life, and activities of daily living for many patients [4].

Accordingly, there has been an increased interest in applying walking performance tests as clinical outcomes in MS. The 6-minute walk (6MW) represents a walking performance test [5] that has received attention as a promising clinical outcome in this population [6-8]. The 6MW appears to provide unique, and perhaps more sensitive, information about the walking performance of persons with MS compared with other tests such as the short distance timed 25-foot walk or the ambulatory steps of the 500-meter walk of the Expanded Disability Status Scale (EDSS) [2].

Some researchers have advocated for a 2-minute walk (2MW) rather than 6MW [9,10] for MS clinical trials. The 2MW is reported to be as valid a measure of walking performance as the 6MW, but it presumably has less strain and demand on the participant and test administrator and therefore appears more feasible. The comparable validity is based on a strong correlation between 2MW and 6MW distances (*r *= 0.94) [10] and similar pattern of correlations with external criteria [9,10]. Importantly, correlations alone might not sufficiently address differences in what the 2MW and 6MW are capturing or the clinical meaningfulness of the observed changes.

Another perspective on identifying the appropriate duration of walking performance tests might involve consideration of physiological variables such as the rate of oxygen consumption (V·O_2_). For example, there is abundant evidence in healthy controls that V·O_2 _increases in a curvilinear manner over the initial 2-3 minutes of submaximal exercise until reaching a plateau or "steady-state" that is maintained over the remainder of submaximal exercise [11,12]. This curvilinear increase in V·O_2 _reflects the lag between energetic supply and demand such that there is greater energy supplied by anaerobic processes than aerobic oxidation during the initiation of submaximal exercise [11,12]. If the period of time before reaching steady-state V·O_2 _extends beyond 2-minutes of submaximal exercise in persons with MS, this would suggest that the 2MW and 6MW represent physiologically different measures of ambulatory performance whose equivalence for MS patients cannot be assumed. The 2MW conceptually would represent a test of aerobic and non-aerobic walking performance, whereas the 6MW would represent a test of aerobic or endurance walking performance.

To date, studies have measured V·O_2 _during the 6MW in persons with MS [13,14], but those studies have typically assumed steady-state V·O_2 _over the last 3 minutes of submaximal exercise and have not documented the actual pattern of change in V·O_2 _over the entire 6MW. Only one study has examined the pattern of change in V·O_2 _over the entire 6MW [15], but this was in a small sample of persons with MS (*N *= 11) and the study did not account for disability status. Disability status has been associated with 6MW performance [6,7] and oxygen cost of walking [14] in MS, and might further impact the magnitude and pattern of change in V O_2 _over the course of the 6MW.

This study examined the pattern of change in V·O_2 _over the course of the 6MW in a large sample of persons with MS. We further examined the possibility that the pattern of change in V·O_2 _over the course of the 6MW might vary as a function of mild, moderate, and severe disability status. Such an examination is important for identifying a physiologic basis for decisions regarding the actual duration of walking performance tests in persons with MS.

## Methods

### Sample

The sample was recruited through referrals from three locally residing neurologists and consisted of 95 persons with clinically-definite MS. The two inclusion criteria were (a) capacity for independent ambulation or ambulation with an assistive device and (b) willingness to complete testing. Persons who had a relapse or underwent administration of steroids in the past 30 days were excluded from participation.

### Procedure

The procedure was approved by a local Institutional Review Board and participants provided written informed consent. The data were collected during a single session in a clinical setting. The participants initially provided demographic information and then underwent a neurological examination for generating an EDSS score [16]. This was followed by attachment of a portable metabolic unit and an accelerometer, and then completion of the 6MW protocol. All participants received $20 remuneration.

### Measures

#### Six-minute walk

The 6MW was performed in a rectangular, carpeted corridor with hallways that exceed 50 m in length and that were clear of obstructions and foot traffic. We provided standardized instructions and emphasized walking as far and as fast as possible for 6 minutes [6]. One researcher followed along side of the participant for safety, while another researcher followed 3 feet behind the participant and recorded the distance travelled (ft) using a measuring wheel (Stanley MW50, New Briton, CT) [7]. If the patient reported a history of falls during ambulation, we placed a gait belt around the person's waist and followed very closely in the event of a slip, trip, or stumble that might cause a fall during the 6MW.

#### Oxygen consumption

V·O_2 _was measured breath-by-breath during the 6MW using a commercially available portable metabolic unit (K4b^2 ^Cosmed, Italy). The O_2 _and CO_2 _analyzers of the portable metabolic unit along with the battery were connected to a harness that was worn on the trunk like a backpack; this portable unit is small (170 × 55 × 100 mm) and light weight (475 g). The flow meter was attached to a mouthpiece that was worn on the participant's face covering both the nose and mouth. The O_2 _and CO_2 _analyzers of the portable metabolic unit were calibrated using verified concentrations of gases, and the flow-meter was calibrated using a 3-L syringe (Hans Rudolph, Kansas City, MO). V·O_2 _was calculated as 30-second averages over the 6MW.

#### Cadence

Steps per minute were measured by an ActiGraph model GT3X accelerometer (Health One Technology, Fort Walton Beach, FL) as a method of monitoring cadence during the 6MW. The accelerometer was worn on an elastic belt around the waist and above the non-dominant hip. The sampling epoch was 1 second. Cadence was calculated as total steps per minute over the 6MW and this device is accurate under controlled conditions in persons with MS [17].

### Data analysis

All data analyses were performed in PASW statistics 18.0. We provided descriptive statistics as mean ± standard deviation (SD) in text and mean ± standard error of the mean (SEM) in Figures. We examined differences in 6MW distance between groups using one-way ANOVA. We examined differences in cadence and V·O_2 _over the 6MW using two-way group by time ANOVA. Post-hoc analyses were performed with an automatic Bonferronni correction of alpha. The effect sizes for the ANOVAs were expressed as eta-squared (η^2^) with values of .01, .06, and .14 indicating small, moderate, and large effects, respectively [18]. We expressed effect sizes for differences in mean as Cohen's d with values of 0.2, 0.5, and 0.8 indicating small, moderate, and large effects, respectively [18].

## Results

### Sample characteristics

The mean age of the sample was 52.8 ± 11.1 yr and the gender distribution was predominantly female (76 women/19 men). The sample primarily had a relapsing-remitting clinical course (n = 78, 82.1% of cases) with the other cases of MS representing secondary progressive (n = 8, 8.4% of cases) and primary progressive (n = 5, 5.3% of cases) clinical courses; there was missing information regarding disease course for 4 (4.2% of cases) participants. The mean disease duration of the sample was 11.9 ± 10.0 yr. Of the 95 persons, 80 or 84.2% were on a disease-modifying therapy and 62 or 65.3% were on a symptomatic therapy. Based on a neurological examination, the median EDSS score was 4.5 with a range between 2.0 and 6.5. This range of EDSS scores permitted formation of three groups consisting of mild (n = 29, EDDS = 2-3.5), moderate (n = 29, EDDS = 4.0-5.5), and severe (n = 37, EDDS = 6.0-6.5) disability consistent with disability benchmarks in epidemiological studies of MS [19].

There was a strong group main effect of disability status on 6MW distance, F(2,92) = 60.25, *p *= .0001, η^2 ^= .58. Those with mild disability walked significantly further than those with moderate (d = 1.30) and severe (d = 2.66) disability, and those with moderate disability walked significantly further than those with severe disability (d = 1.44). The mean 6MW distances for those with mild, moderate, and severe disability were 1723 ± 252 ft, 1389 ± 264 ft, and 979 ± 300 ft, respectively. Interestingly, the coefficients of variation (100 × SD/M) for mild, moderate, and severe disability were 15%, 19%, and 31%, respectively.

**Figure 1:**
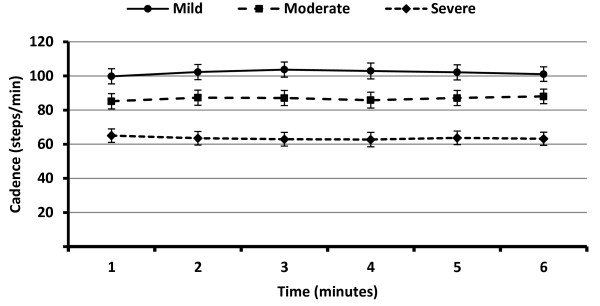
**Cadence (steps/minute) over the six-minute walk test for persons with mild (n = 29), moderate (n = 29), and severe (n = 37) disability scores**.

There was a strong group main effect on cadence (steps/minute) during the 6MW, F(2,91) = 22.82, *p *= .0001, η^2 ^= .33. Those with mild disability took significantly more steps per minute during the 6MW than those with moderate (d = 0.66) and severe (d = 1.64) disability, and those with moderate disability took significantly more steps per minute during the 6MW than those with severe disability (d = 0.99). This is illustrated in Figure 1. Cadence did not differ over the 6MW by time, F(5,455) = 0.42, *p *= .84, η^2 ^= .01, or group and time, F(10,455) = 1.08, *p *= .37, η^2 ^= .02.

**Figure 2:**
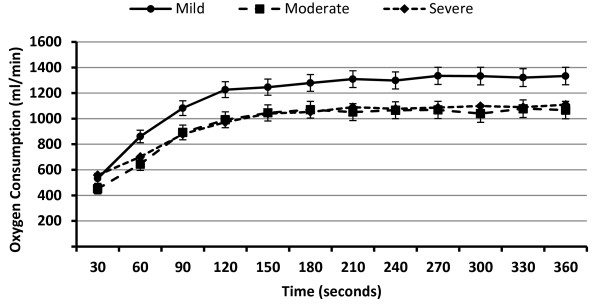
**Oxygen consumption (milliliters/minute) over the six-minute walk test for persons with mild (n = 29), moderate (n = 29), and severe (n = 37) disability scores**.

There was a very strong time main effect on V·O_2 _during the 6MW, F(11,1012) = 357.58, *p *= .0001, η^2 ^= .80, as well as a moderate group main effect, F(2,92) = 4.41, *p *= .015, η^2 ^= .09, and a moderate group by time interaction, F(22,1012) = 4.66, *p *= .0001, η^2 ^= .09. Overall, V·O_2 _increased significantly every 30 seconds over the first 3 minutes of the 6MW, and then remained stable over the second 3 minutes of the 6MW; this is illustrated in Figure 2. The overall pattern of change in V·O_2 _over the 6MW was not changed in additional analyses that controlled for the presence/absence of disease-modifying or symptomatic therapy. The rate of increase in V·O_2 _was steeper in those with mild disability than those with moderate and severe disability based on the interaction, and those with mild disability had a higher rate of V·O_2 _than those with moderate and severe disability based on the group main effect.

## Discussion

Researchers have recently advocated for a 2MW rather than a 6MW as a walking performance test in persons with MS, and this is based on a strong correlation between distances and similar correlations with external criteria [9,10]. This evidence alone is not sufficient for selecting a single test duration given the likely different physiological and metabolic demands of the 2MW and 6MW and the heterogeneous disability of individual MS patients. Accordingly, for this study we adopted a physiological perspective for understanding the appropriate duration of a walking performance test in MS, and we provide data on the pattern of change in V·O_2 _over the 6MW at various degrees of disability using the EDSS as gold standard. V·O_2 _changed in a curvilinear pattern during the first 3 minutes of the 6MW, and then reached a steady-state characterized by no further changes in V·O_2 _over the remainder of the 6MW. Such a pattern of change in V·O_2 _occurred despite similar pacing over the entire 6MW based on cadence measured by an accelerometer. This is explained by the lag in aerobic oxidation during the initiation of steady-state, submaximal exercise [11,12]. Overall, the pattern of change in V·O_2 _suggests that there are different metabolic systems providing energy for ambulation during phases of the 6MW. Both aerobic and anaerobic systems are providing energy for walking in the first part of the 6MW, whereas aerobic metabolism is the primary energy system used for walking during the second part of the 6MW. This observation might be further examined by administering tests of anaerobic and aerobic power and examining the association with 2MW and 6MW performance in persons with MS. Anaerobic power could be measured using a Wingate Test (i.e., pedal a manually braked bicycle ergometer for 30 seconds with an all out pace), whereas aerobic power could be measured using a maximal, incremental exercise test (i.e., pedal an electronically braked ergometer over 10-12 minute period with increasing resistance until volitional termination). Such tests can be administered in persons with MS and would provide further information on the physiological and metabolic systems underlying 2MW and 6MW performance. This would be important for identifying the possibility that these tests measure different features of walking and therefore have different interpretations or applications as clinical outcome variables.

We compared our data on the pattern of change in V·O_2 _over the 6MW with previous research involving persons with MS [15]. That previous research included a small sample of persons with MS (n = 11) and reported that V·O_2 _increased over the first 3 minutes of the 6MW and then reached steady-state over the remainder of the test. This pattern of change was consistent with that observed during submaximal exercise in non-diseased, healthy adults [11,12], but the level of increase in V·O_2 _during the 6MW is higher in controls than persons with MS [15]; this is expected based on the difference in walking speed, but has not been examined across levels of disability status in MS versus controls. We confirmed that V·O_2 _increases over the first 3 minutes of the 6MW and then reaches "steady state," but in a nearly 10 times larger sample of persons with MS. We further reported on the novel result that the pattern of change over the first 3 minutes was generally consistent across mild, moderate, and severe disability status, although the slope was steeper and the absolute level of V·O_2 _was higher in those with mild disability compared with moderate and severe disability. This is caused, in part, by the difference in walking speed and its associated effect on V·O_2_. In particular, if we expressed V·O_2 _relative to walking speed (i.e., oxygen cost of walking), there would be a difference between the moderate and severe disability groups given similar energy expenditure, but different walking speed. Nevertheless, there appears to be consistent evidence for a curvilinear pattern of change in V·O_2 _over the first 3 minutes of the 6MW in MS, and this has implications for identifying the primary source of energy for walking during the 2MW and 6MW. The pattern of change in V·O_2 _would suggest that the 2MW represents a test of both aerobic and anaerobic aspects of walking performance, whereas the 6MW represents a test of primarily aerobic or endurance aspects of walking performance in MS. Conceptually, 2MW performance might be associated with everyday tasks that require brief, but intense bouts of ambulation (e.g., stair climbing or crossing the street), whereas 6MW performance might be associated with everyday tasks that require sustained, submaximal bouts of ambulation (e.g., grocery shopping).

We compared our data on the 6MW with those of previous research [6] for further understanding the veracity of this performance test for differentiating disability status in MS. That previous research [6] observed a reduction in walking distance (mean ± standard deviation in ft) across three levels of disability status, namely mild (1958 ± 155 ft; EDSS = 0-2.5; n = 15), moderate (1636 ± 332 ft; EDSS = 3-4; n = 19), and severe (1254 ± 259 ft; EDSS = 4.5-6.5; n = 6) disability. We observed a similar reduction in walking distance across the three levels of disability status of mild (1723 ft; EDSS = 2-3.5; n = 29), moderate (1389 ft; EDSS = 4-5.5; n = 29), and severe (979 ft; EDSS = 6.0-6.5; n = 37) disability. Interesting, the coefficient of variability was larger for persons with moderate and severe disability than mild disability in the present study and previous research [6]. We further note that all participants walked for the entire 6-minute period, and we had no falls or other adverse events. This too is similar with the observations of previous research [6]. Overall, we replicated the capacity of the 6MW for differentiating disability status, particularly in the severe group that had a small sample size in previous research [6], and recommend that the 6MW continue to be considered as a performance assessment of walking endurance in MS as it is in other neurological conditions such as Parkinson's disease [20,21].

We measured cadence during the 6MW using an accelerometer as an initial method of examining spatiotemporal parameters of pacing; this has not been undertaken in previous research. Interestingly, we observed that cadence (i.e., steps per minute) did not change over the 6MW for any of the groups who differed in disability status, but it did differ significantly among the three groups. Those with mild disability had a greater cadence during the 6MW than those with moderate and severe disability, and those with moderate disability had a greater cadence during the 6MW than those with severe disability. This indicates that the spatiotemporal parameters of pacing do not change over the 6MW in persons with MS (i.e., persons select and maintain a stable cadence over the entire 6MW) perhaps to optimize energy expenditure, and future researchers might consider other temporal and spatial parameters of gait such as step length during the 6MW given that there has been an observation of possible differences in minute-by-minute walking speeds during the 6MW across disability status [6]. We note that there might be different neural systems controlling step rate and length in MS, and perhaps this is important for monitoring in clinical trials of MS. Of note, the sample in the present study has a different disability composition than that of the previous publication examining changes in performance pattern during the 6MW [6]. This issue could be further explored through application of devices such as the Intelligent Device for Energy Expenditure and Physical Activity or IDEEA^® ^or GAITRite™ electronic walkway and might further our understanding of gait parameters that are associated with long distance walking performance and identify targets of rehabilitation for improving this valued function.

We did not administer an independent 2MW in this study and have no information on V·O_2 _over the actual 2MW test. Nevertheless, we suspect that V·O_2 _would actually be similar during the 2MW compared with the first 2 minutes of the 6MW. This is based on the similar distance walked during the 2MW and first 2 minutes of the 6MW in persons with MS [10]. By extension, walking speed would be comparable between the 2MW and first 2 minutes of the 6MW and result in a similar metabolic demand. This observation is important as it minimizes the concern that our results regarding change in V·O_2 _over the initial part of the 6MW are not applicable for the 2MW. However, even if the change in V·O_2 _was not applicable it would not undermine our argument that there are different metabolic processes underlying the 2MW and 6MW, as one would expect a steeper slope of the V·O_2 _response if the intensity was actually greater when performing the 2MW test. Nevertheless, such assumptions should be confirmed in subsequent research along with the recommendation of administering tests of anaerobic and aerobic power in MS.

There are notable strengths of the current study including the standardized administration of the 6MW protocol, measurement of V·O_2 _and cadence over the 6MW, and relatively large sample of persons with MS and subsamples with a proportionate range of disability status. The limitations include (a) lack of a control group for further comparing V·O_2_, cadence, and distance over the 6MW, (b) lack of measurement of V·O_2 _and cadence over the 2MW, (c) the cross-sectional design and lack of information on the comparative sensitivity and responsiveness for capturing change in 2MW and 6MW performance over time and in the context of an intervention trial, (d) lack of external criteria for validating the 2MW and 6MW against anaerobic and aerobic power, and (e) lack of control for fatigue levels and exact types of symptomatic therapies that might impact ambulation, V·O_2_, and cadence.

## Conclusion

We provide new data on V·O_2 _and cadence over the 6MW across disability status, and the data on V·O_2 _over the 6MW, in particular, has implications for decisions regarding the domain of long distance walking to be captured in future applications of the 2MW and 6MW tests in MS and perhaps other neurological conditions.

## Competing interests

RWM and MGD both have consulting contracts with Biogen IDEC and Acorda Therapeutics. This investigation was directly supported by a grant from the OSF Foundation Hospital and an investigator initiated grant from Biogen IDEC.

## Authors' contributions

RWM was responsible for the conception and design of the study, acquisition of data, data analysis and interpretation, drafting and revising the manuscript, and final approval of the manuscript. YS and SB were responsible for the design of the study, acquisition of data, data analysis, revising the manuscript, and final approval of the manuscript. BMS and JP were responsible for the acquisition of data, data interpretation, revising the manuscript, and final approval of the manuscript. JJS, MDG, and BF were responsible for data interpretation, revising the manuscript, and final approval of the manuscript. All authors have read and approved the final manuscript.

## Pre-publication history

The pre-publication history for this paper can be accessed here:

http://www.biomedcentral.com/1471-2377/12/6/prepub
